# Tanner’s Manual of Clinical Medicine

**Published:** 1855-09

**Authors:** 


*Tanner's Manual of Clinical Medicine.* Blanchard & Lea, Philadelphia.

We have received this choice little work, which is well calculated to answer a requirement on the part of the profession. It is intended for daily reference, and to aid in the study of disease at the bed-side. The first section is upon the "faculty of observa tion," the second, "the general conduct of the practitioner of medicine," the third, the "clinical examination of the patient," &c., and finally closes with the "Code of Ethics adopted by the American Medical Association."

This valuable aid to the student and practitioner is so small as to be carried readily in the pocket, and while perusing it the thought occurred to us how profitably the time of the practitioner could be employed in its perusal when detained away from his home in a case which did not require his immediate attention. That the hours may not drag heavily, he is oftimes forced to employ them upon some of the light valueless productions now so largely published and generally read. In "Tanner's Manuel," however, he will find much of substantial value and worthy of remembrance.

